# Supplementary material for: Deletion of ptn1, a PTEN/TEP1 Orthologue, in Ustilago maydis Reduces Pathogenicity and Teliospore Development
Source: J Fungi (Basel). 2018 Dec 20;5(1):1. doi: 10.3390/jof5010001 (PMC6462984; doi:10.3390/jof5010001)
Supplement: Supplementary file 1 [file jof-05-00001-s001.pdf]

Supplementary materials

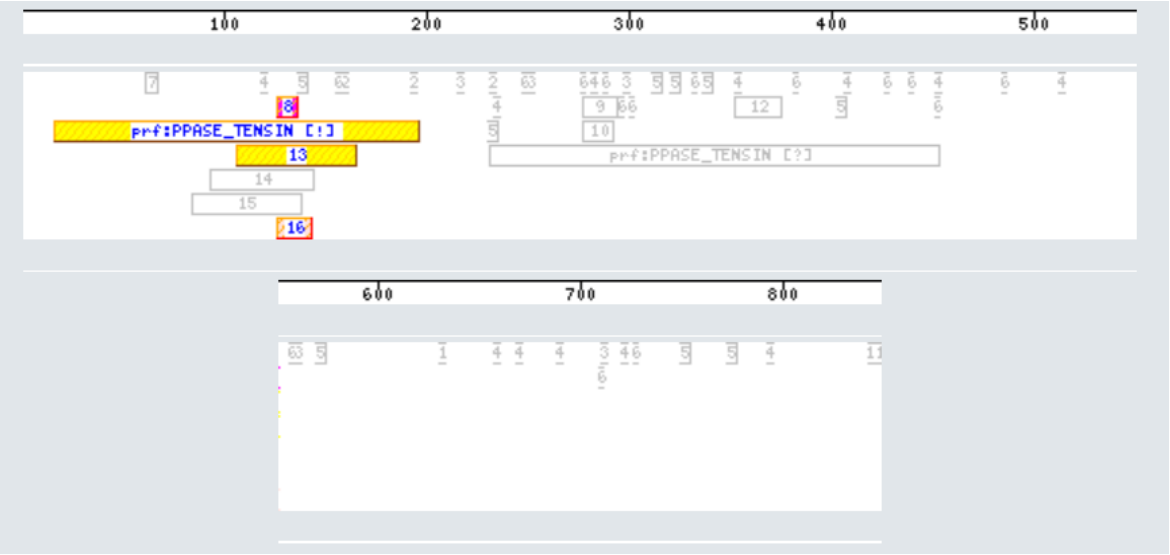

Figure S1. Motif scan analysis of Ptn1 using Hits (https://myhits.isb-sib.ch/) indicated the presence of Phosphatase tensin-type domain profile.

```
# SignalP-4.1 euk predictions
>XP_011390213.1  hypothetical protein UMAG_03760 _Ustilago maydis 521_
```

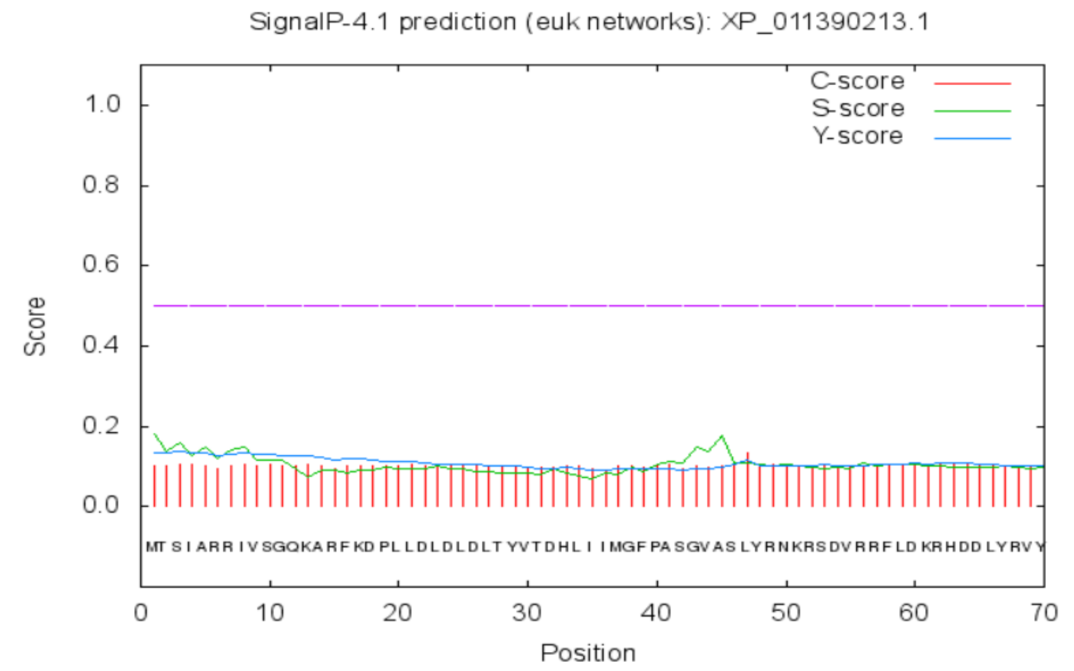


```
# Measure  Position  Value    Cutoff   signal peptide?
  max. C    47       0.135
  max. Y    13       0.128
  max. S     1       0.179
  mean S     1-12    0.134
       D     1-12    0.131   0.450   NO
Name=XP_011390213.1     SP='NO' D=0.131 D-cutoff=0.450 Networks=SignalP-noTM
```

Figure S2. SignalP 4.1 prediction of the presence signal peptide. SignalP could not find a signal peptide in Ptn1.

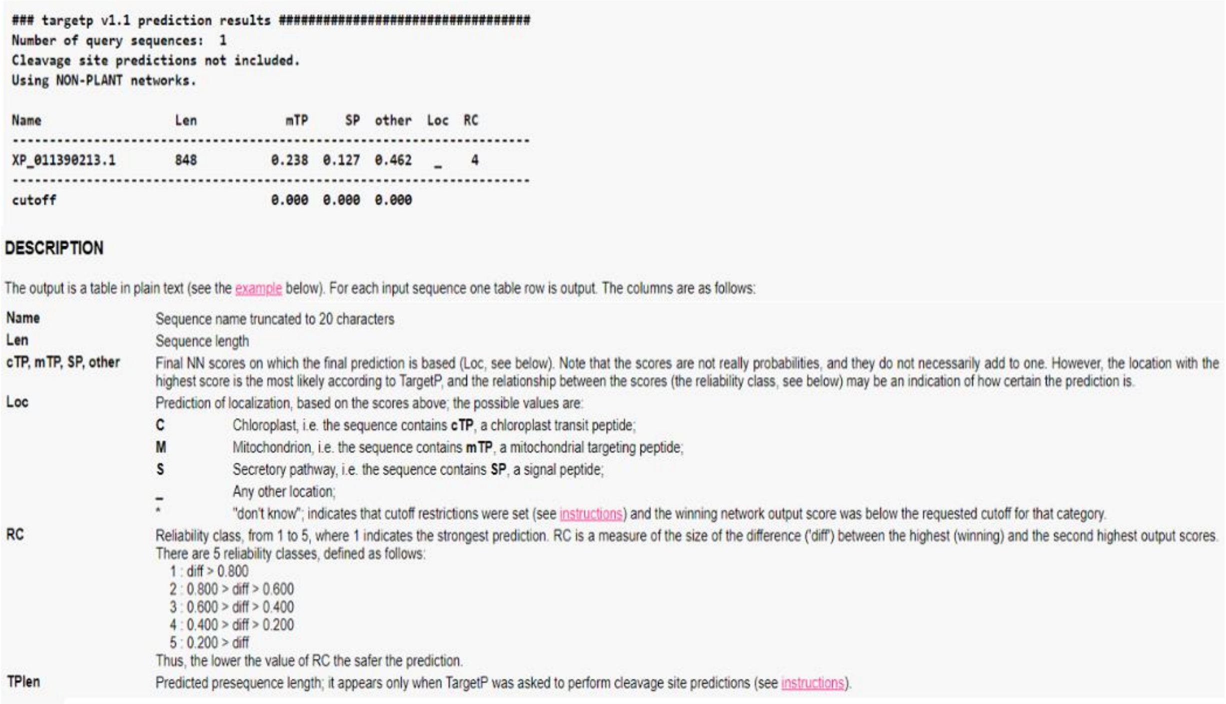

```
### targetp v1.1 prediction results ##################################
Number of query sequences:  1
Cleavage site predictions not included.
Using NON-PLANT networks.

Name                  Len            mTP     SP  other  Loc  RC
----------------------------------------------------------------------
XP_011390213.1        848          0.238  0.127  0.462   _    4
----------------------------------------------------------------------
cutoff                             0.000  0.000  0.000
```

DESCRIPTION

The output is a table in plain text (see the example below). For each input sequence one table row is output. The columns are as follows:

**Name**	Sequence name truncated to 20 characters
**Len**	Sequence length
**cTP, mTP, SP, other**	Final NN scores on which the final prediction is based (Loc, see below). Note that the scores are not really probabilities, and they do not necessarily add to one. However, the location with the highest score is the most likely according to TargetP, and the relationship between the scores (the reliability class, see below) may be an indication of how certain the prediction is.
**Loc**	Prediction of localization, based on the scores above; the possible values are:<br>**C** Chloroplast, i.e. the sequence contains **cTP**, a chloroplast transit peptide;<br>**M** Mitochondrion, i.e. the sequence contains **mTP**, a mitochondrial targeting peptide;<br>**S** Secretory pathway, i.e. the sequence contains **SP**, a signal peptide;<br>**_** Any other location;<br>* "don't know"; indicates that cutoff restrictions were set (see instructions) and the winning network output score was below the requested cutoff for that category.
**RC**	Reliability class, from 1 to 5, where 1 indicates the strongest prediction. RC is a measure of the size of the difference ('diff') between the highest (winning) and the second highest output scores. There are 5 reliability classes, defined as follows:<br>1 : diff > 0.800<br>2 : 0.800 > diff > 0.600<br>3 : 0.600 > diff > 0.400<br>4 : 0.400 > diff > 0.200<br>5 : 0.200 > diff<br>Thus, the lower the value of RC the safer the prediction.
**TPlen**	Predicted presequence length; it appears only when TargetP was asked to perform cleavage site predictions (see instructions).

Figure S3. TargetP prediction of sub-cellular localization of Ptn1. TargetP analysis indicated that Ptn1 is not a secreted protein and may have a sub-cellular location other than mitochondria.

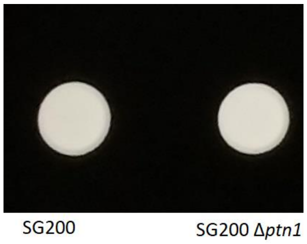

Figure S4. The charcoal assay for fuzz production performed with SG200 and SG200 Δ*ptn1*strains. No differences were observed in the ability to produce aerial hyphae.

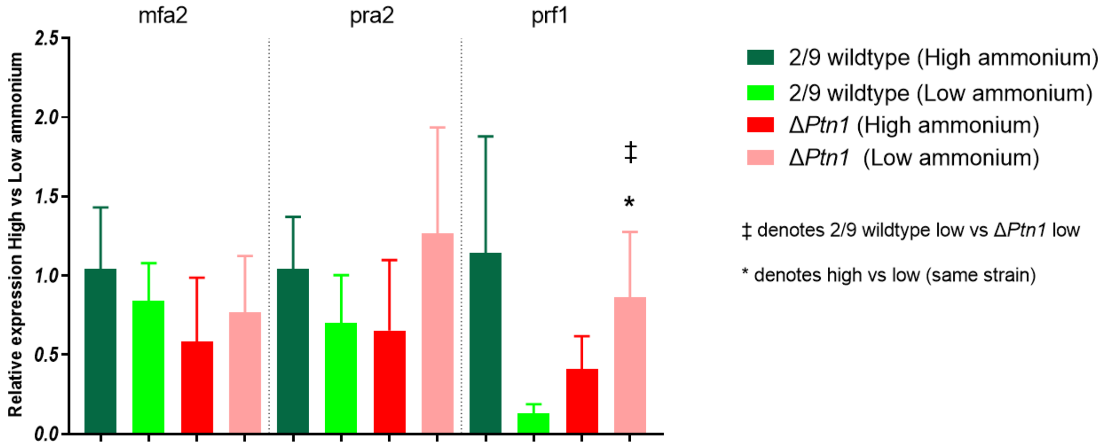

Figure S5. The relative expression analysis of *mfa2*, *pra2*, and *prf1* (*U. maydis* mating pathway genes) in wildtype and *Δptn1* under high and low ammonium conditions. The RNA expression data were analyzed in GraphPad Prism using unpaired T-test after normalization to wild type expression under high ammonium conditions; a probability value of $P < 0.05$ was considered as a significant difference and is indicated by *.

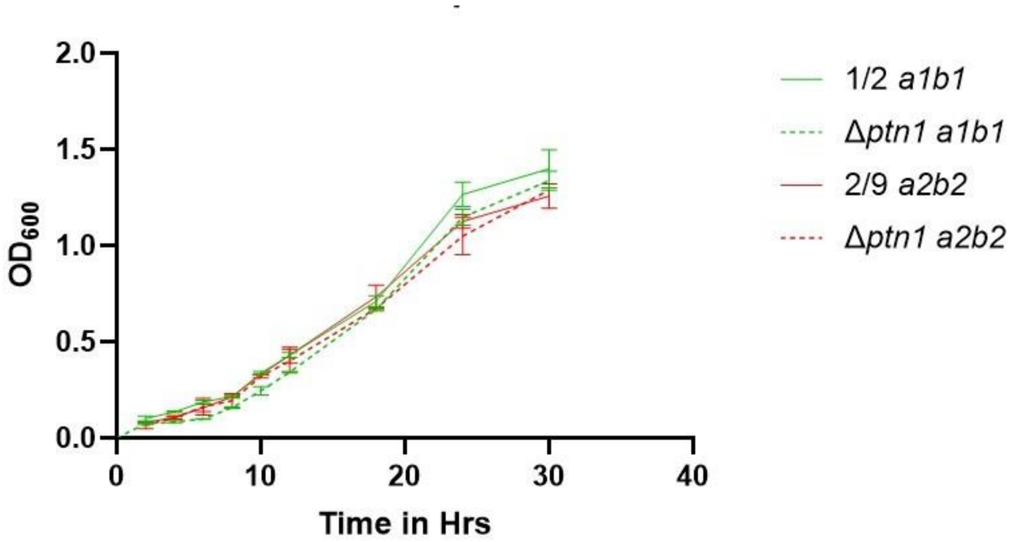

Figure S6: Growth rates of 1/2, 2/9, 1/2 Δ*ptn1* and 2/9 Δ*ptn1* strains grown in YPS media were measured by OD_{600} at an initial interval of 2 h until 12 hours and then every 6 hours until 30 hours. The data did not show any significant differences in growth rates.

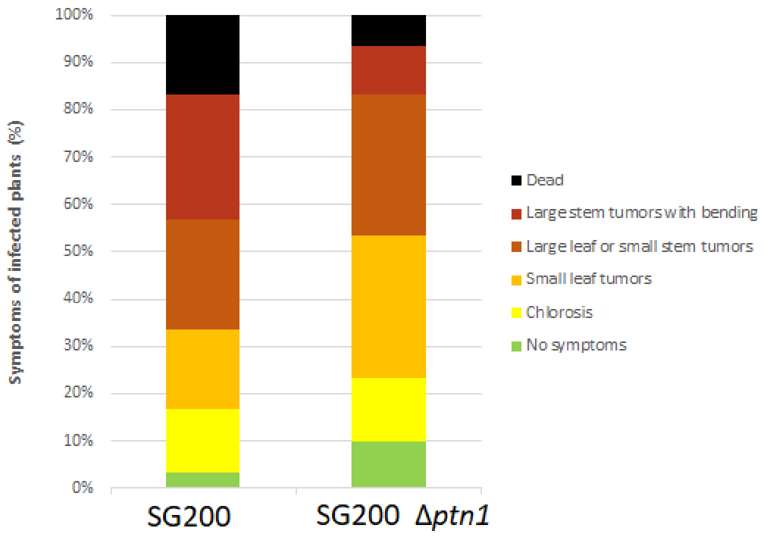

Figure S7: Infection of plants with Δ*ptn1* deletion strains in SG200 background. The deletion strain SG200 Δ*ptn1* showed a reduction in pathogenicity compared to wildtype SG200 (nearly significant, *P*= 0.05048, Kruskal-Wallis multiple comparison test*)*

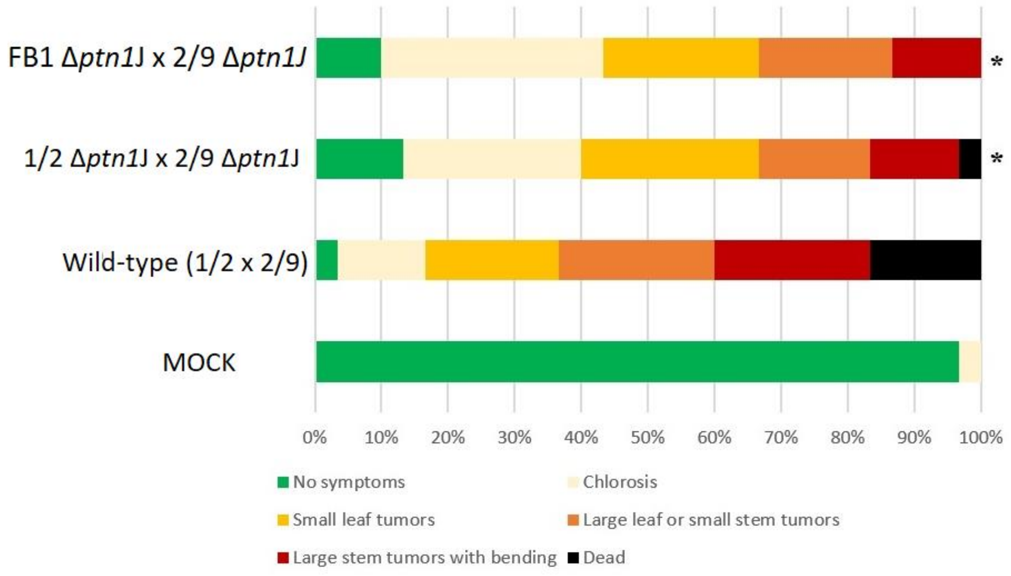

Figure S8. Infection of plants with Δ*ptn1* deletion strains in different backgrounds and created through two different methods. Similar reductions in pathogenicity were seen, regardless of the method used to generate the deletions or the genetic backgrounds of the compatible mating partners. 1/2 Δ*ptn1*J and 2/9 Δ*ptn1*J strains generated using the overlap- PCR method [27,28] were found to be less infectious compared to WT (1/2 and 2/9). Pathogenicity was similarly impaired for infections with strains FB1 Δ*ptn1*J x 2/9 Δ*ptn1*; FB1 Δ*ptn1* was generated via the overlap method, while 2/9 Δ*ptn1* as produced with the DelsGate method.

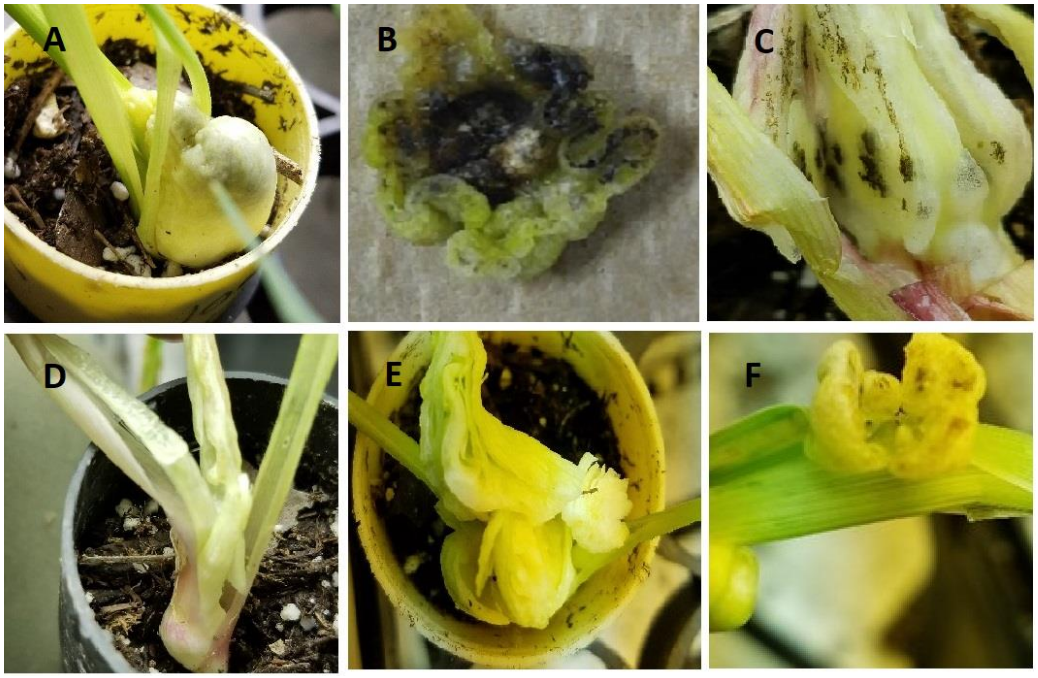

Figure S9. Teliospores from infected maize plants (Clockwise): A, B and C represent tumors from plants infected with both wild type partners 1/2 x 2/9 (*a1b1* x *a2b2*). D, E, F represent tumors of plants from Δ*ptn1* (*a1b1* x *a2b2*) infection. WT (1/2 x 2/9) infected plants produced normal tumors which were filled with black teliospores, whereas mature black teliospores were not clearly visible in most of the tumors produced from Δ*ptn1* infected plants.

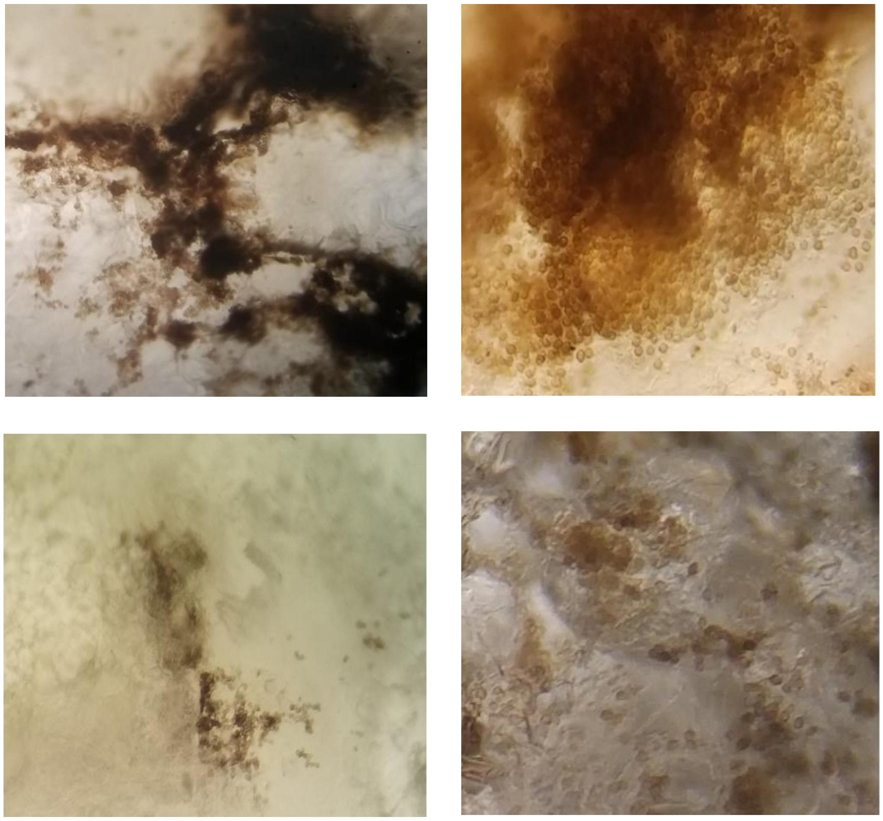

Figure S10. Dissection of tumors under microscope showing teliospores. Upper panels represent spores from WT 1/2 x WT 2/9 infected plants and lower panels show spores from Δ*ptn1* infected plants. WT tumors contained visibly more teliospores.

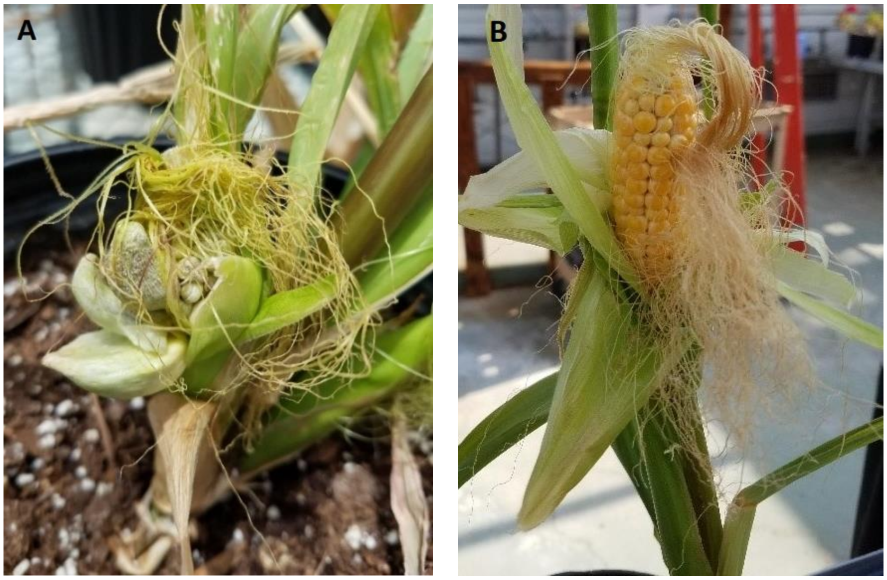

Figure S11. Deletion of *ptn1* alters ability to produce tumors in maize cobs. The deletion strains were not able to produce tumors with teliospores when infection was performed on maize ears (A) WT (1/2) × WT (2/9), (B) Δ*ptn1*× Δ*ptn1* in the same respective genetic backgrounds as in (A).

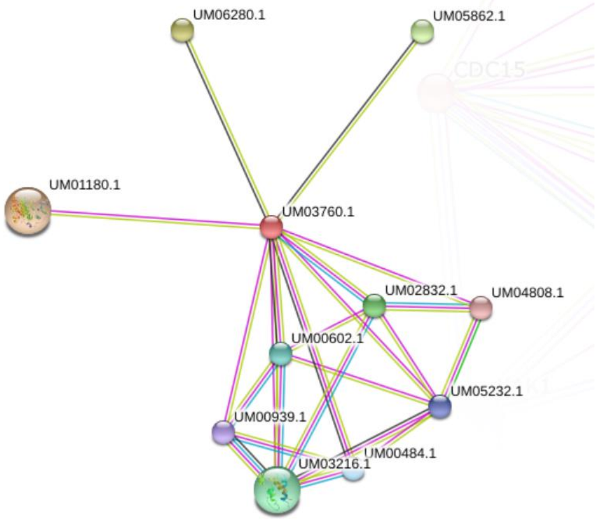

Figure S12. Interaction Network Analysis of Ptn1 protein sequence using STRING software (https://string-db.org) indicated one of the potential interactors as UM02832 (Ras1 protein) of *U. maydis*

**Table S1. A**. Aligned Proteins			
Protein Name	Identity	Expected Value	Matched Length
A0A0D1E0C2_USTMA	1	0	848
R9NVM2_PSEHS	0.76	e-163	850
I2FUC9_USTH4	0.69	e-158	853
A0A1K0H8X3_9BASI	0.69	e-156	853
E6ZMB4_SPORE	0.68	e-144	852
W3VTN5_PSEA5	0.67	e-162	860
M9LVY0_PSEA3	0.67	e-162	860
A0A0F7S2A1_9BASI	0.67	e-110	697
A0A081CHB7_PSEA2	0.66	e-159	851
A0A127ZGP6_9BASI	0.66	e-150	857
A0A077QWF3_9BASI	0.65	e-159	859
V5EYU7_KALBG	0.45	1.00E-96	844
TPTE2_MACFA	0.38	1.00E-42	193
TPTE2_HUMAN	0.38	7.00E-42	193
PTEN1_ARATH	0.38	4.00E-36	189
A0A0D2PH34_9AGAR	0.37	1.00E-31	311
TPTE_HUMAN	0.37	2.00E-40	193
PTN2B_ARATH	0.36	1.00E-38	204
A0A0C9WXR8_9AGAR	0.35	1.00E-33	340
A0A066VX13_9BASI	0.34	9.00E-90	877
CNRN_DICDI	0.32	1.00E-37	237
PTEN_MOUSE	0.31	2.00E-46	251
PTEN_HUMAN	0.31	2.00E-46	251
PTEN_CANLF	0.31	2.00E-46	251
PTEN_DICDI	0.31	4.00E-44	237
PTEN_XENLA	0.31	2.00E-42	239
5bzz_D Chain(D)	0.3	6.00E-40	238
5bzz_C Chain(C)	0.3	6.00E-40	238
5bzz_B Chain(B)	0.3	6.00E-40	238
5bzz_A Chain(A)	0.3	6.00E-40	238
5bzx_D Chain(D)	0.3	6.00E-40	238
5bzx_C Chain(C)	0.3	6.00E-40	238
5bzx_B Chain(B)	0.3	6.00E-40	238
5bzx_A Chain(A)	0.3	6.00E-40	238
1d5r_A Chain(A)	0.3	6.00E-40	238
5bug_D Chain(D)	0.3	6.00E-40	238
5bug_C Chain(C)	0.3	6.00E-40	238

5bug_B Chain(B)	0.3	6.00E-40	238
5bug_A Chain(A)	0.3	6.00E-40	238
3v0h_B Chain(B)	0.3	7.00E-36	240
3v0f_A Chain(A)	0.3	1.00E-35	240
3v0g_C Chain(C)	0.3	4.00E-35	239
3v0g_A Chain(A)	0.3	4.00E-35	239
3v0h_A Chain(A)	0.3	4.00E-35	239
3v0d_A Chain(A)	0.3	1.00E-34	238
3v0f_B Chain(B)	0.3	1.00E-34	238
K5WK49_AGABU	0.29	8.00E-36	423
3v0g_B Chain(B)	0.29	3.00E-36	249
3v0d_B Chain(B)	0.29	3.00E-34	243
3v0g_D Chain(D)	0.29	3.00E-34	243
A0A0C3CDL4_HEBCY	0.28	4.00E-31	444
A0A177VUH2_9BASI	0.26	4.00E-54	733
A0A177VHL6_9BASI	0.26	4.00E-54	733
W4JQV9_9HOMO	0.26	9.00E-51	616
A0A1J7GYH2_LUPAN	0.24	7.00E-48	518
A0A166LBZ8_9HOMO	0.23	8.00E-61	767
PTN2A_ARATH	0.22	1.00E-38	463
A0A0C9WXR8_9AGAR	0.18	8.00E-52	563
A0A0D2PH34_9AGAR	0.18	3.00E-51	567
A0A0C3CDL4_HEBCY	0.17	8.00E-49	572
A0A087UC66_9ARAC	0.16	4.00E-49	775
TENS3_HUMAN	0.16	6.00E-40	792
PTEN1_ARATH	0.16	6.00E-04	219
TENS3_MOUSE	0.16	6.00E-34	769
A0A177VUH2_9BASI	0.15	1.00E-16	555
A0A177VHL6_9BASI	0.15	1.00E-16	555
K5WK49_AGABU	0.15	7.00E-48	607
K1RBC4_CRAGI	0.14	4.00E-49	768

Table S1. B. Matched Protein Structures

Protein Name	Identity	Expected Value	Matched Length
5bzz_D Chain(D)	0.3	6.00E-40	238
5bzz_C Chain(C)	0.3	6.00E-40	238

5bzz_B Chain(B)	0.3	6.00E-40	238
5bzz_A Chain(A)	0.3	6.00E-40	238
5bzx_D Chain(D)	0.3	6.00E-40	238
5bzx_C Chain(C)	0.3	6.00E-40	238
5bzx_B Chain(B)	0.3	6.00E-40	238
5bzx_A Chain(A)	0.3	6.00E-40	238
1d5r_A Chain(A)	0.3	6.00E-40	238
5bug_D Chain(D)	0.3	6.00E-40	238
5bug_C Chain(C)	0.3	6.00E-40	238
5bug_B Chain(B)	0.3	6.00E-40	238
5bug_A Chain(A)	0.3	6.00E-40	238
3v0h_B Chain(B)	0.3	7.00E-36	240
3v0f_A Chain(A)	0.3	1.00E-35	240
3v0g_C Chain(C)	0.3	4.00E-35	239
3v0g_A Chain(A)	0.3	4.00E-35	239
3v0h_A Chain(A)	0.3	4.00E-35	239
3v0d_A Chain(A)	0.3	1.00E-34	238
3v0f_B Chain(B)	0.3	1.00E-34	238
3v0g_B Chain(B)	0.29	3.00E-36	249
3v0d_B Chain(B)	0.29	3.00E-34	243
3v0g_D Chain(D)	0.29	3.00E-34	243

Table. S1 Protein prediction analysis of Ptn1 using PredictProtein (https://www.predictprotein.org). (A) Shows the list of aligned proteins by primary amino acid sequence. (B) Shows the list of matched protein structures.
